# Edible mycelium as proliferation and differentiation support for anchorage-dependent animal cells in cultivated meat production

**DOI:** 10.1038/s41538-024-00263-0

**Published:** 2024-04-30

**Authors:** Minami Ogawa, Alex S. Kermani, Mayrene J. Huynh, Keith Baar, J. Kent Leach, David E. Block

**Affiliations:** 1 Department of Food Science and Technology, University of California, Davis, Davis, CA 95616 USA; 2 Department of Materials Science and Engineering, University of California, Davis, Davis, CA 95616 USA; 3 Department of Neurobiology, Physiology, and Behavior, University of California, Davis, Davis, CA 95616 USA; 4 Department of Orthopaedic Surgery, UC Davis Health, Sacramento, CA 95817 USA; 5 Department of Biomedical Engineering, University of California, Davis, Davis, CA 95616 USA; 6 Department of Chemical Engineering, University of California, Davis, Davis, CA 95616 USA; 7 Department of Viticulture and Enology, University of California, Davis, Davis, CA 95616 USA

**Keywords:** Cell growth, Cell adhesion, Fungi

## Abstract

Cultivated meat production requires bioprocess optimization to achieve cell densities that are multiple orders of magnitude higher compared to conventional cell culture techniques. These processes must maximize resource efficiency and cost-effectiveness by attaining high cell growth productivity per unit of medium. Microcarriers, or carriers, are compatible with large-scale bioreactor use, and offer a large surface-area-to-volume ratio for the adhesion and proliferation of anchorage-dependent animal cells. An ongoing challenge persists in the efficient retrieval of cells from the carriers, with conflicting reports on the effectiveness of trypsinization and the need for additional optimization measures such as carrier sieving. To surmount this issue, edible carriers have been proposed, offering the advantage of integration into the final food product while providing opportunities for texture, flavor, and nutritional incorporation. Recently, a proof of concept (POC) utilizing inactivated mycelium biomass derived from edible filamentous fungus demonstrated its potential as a support structure for myoblasts. However, this POC relied on a model mammalian cell line combination with a single mycelium species, limiting realistic applicability to cultivated meat production. This study aims to advance the POC. We found that the species of fungi composing the carriers impacts C2C12 myoblast cell attachment—with carriers derived from *Aspergillus oryzae* promoting the best proliferation. C2C12 myoblasts effectively differentiated on mycelium carriers when induced in myogenic differentiation media. Mycelium carriers also supported proliferation and differentiation of bovine satellite cells. These findings demonstrate the potential of edible mycelium carrier technology to be readily adapted in product development within the cultivated meat industry.

## Introduction

Large-scale culture of anchorage-dependent animal cells is necessary for regenerative medicine, tissue engineering, and biopharmaceutical manufacturing. However, if cellular agriculture is to meet the rising need for animal protein, it must scale orders of magnitude beyond established methods^1^. In addition, achieving a high productivity of cell growth per unit of medium is essential to maximize resource efficiency and reach price parity with conventional meat^2–4^. To address the scalability and productivity bottlenecks, microcarriers can be used to achieve higher cell densities for anchorage-dependent cells^5^^,6^. Microcarriers are suspended scaffolds commonly used in bioreactors or stirred tank reactors. They act as physical supports that provide a surface for cells to adhere and proliferate on^7–10^. The microcarriers remain suspended in medium under gentle agitation, allowing adherent cells to be cultivated in suspension and offering a large surface-area-to-volume ratio to support high cell-density cultures relative to standard 2D planar cell culture practices^11,12^. In addition, the growth surface can easily be expanded by “bead-to-bead transfer,” where cells on microcarriers can migrate to newly added microcarriers in a near-confluent culture and cells maintain a longer exponential growth phase^13^. Microcarriers also allow a smooth transition from 2D surface growth to 3D culture and can be adapted for use in a variety of culture vessel geometries and existing cell culture infrastructure. Therefore, compared to other culturing methods like aggregates and fixed bed reactors, microcarriers are easier to control, monitor, implement, and are more cost-effective—leading to reduced training, capital expenditure, and setup costs^14^.

Current commercial microcarriers are mostly composed of nonedible synthetic materials (e.g., polystyrene, cross-linked dextran, cross-linked cellulose, and high-density polyethylene silica) and/or animal-derived components. These microcarriers are optimized for use in cell culture to produce monoclonal antibodies, vaccines, and proteins of interest^15^. Few applications focus on maximizing cell mass. A key challenge of these microcarriers is cell harvesting. The efficiency of trypsinization remains controversial, and additional microcarrier sieving procedures are needed to improve cell retrieval from the carriers^16,17^. Furthermore, harvesting at the 200-250 m^3^ scale required for cultivated meat^2^ is likely not practical and will generate large amounts of solid waste in the form of used synthetic microcarriers or introduce hazards such as microplastic ingestion if not completely removed from the final product^2^. To circumvent this issue, carriers can be composed of edible or dissolvable material. Dissolvable carriers are made of degradable materials (e.g., denatured collagen, cross-linked polygalacturonic acid, alginate, etc.) that can be dissolved once the cells on microcarriers have reached confluency^18,19^. However, like the synthetic carriers, dissolving operations add complexity and cost to cell culture and degradation must be carefully controlled to retain cell function. Edible carriers, on the other hand, can be incorporated into a final product, simplifying the bioprocess by eliminating any dissociation or dissolving steps and adding to the gross mass yield. For food applications like cultivated meat, this provides an additional avenue to incorporate texture, flavor, and nutrition^20^. The discovery of edible materials that support proliferation and differentiation of cells is crucial, especially for the sustainable food technology sector, where food-grade requirements must be met. However, there is a research gap pertaining to edible materials compatible with cell culture. There are very few edible carriers that are readily available commercially, and for those that are available, data on cell proliferation and differentiation are scarce.

Recently, we provided proof of concept (POC) that edible, inactive biomass of *Aspergillus oryzae* can function as a support for myoblast cells^21,22^. *Aspergillus oryzae is* a Generally Recognized As Safe (GRAS) fungal species commonly used in food fermentation and its biomass is consumed as an alternative protein (known as mycoprotein)^23–25^. Filamentous fungi are fast growing, robust microorganisms that can grow on simple, low-cost carbon sources, including agricultural waste^18^. These important properties can contribute to reducing the overall cost of cultivated meat products and increase sustainability. The morphological and biochemical properties of these fungi can be tuned by fermentation bioprocesses, with know-how that has already been optimized for large scale fermentation vessels^26,27^. Mycoprotein contains all essential amino acids and has a protein digestibility-corrected amino acid score of 0.996, making it a complete protein source with bioavailability similar to that of dairy milk and better than wheat-based or soy-based protein^28–30^. Therefore, edible filamentous fungi are an ideal, sustainable biomaterial for cultivated meat microcarriers.

Previous work on mycelium carriers supporting C2C12 attachment and growth was limited by investigating biocompatibility of a single edible fungal strain and a model animal cell line^21,22^. In this study, we expand on the POC and hypothesize that mycelium can be a carrier for animal cell growth and differentiation by assessing cell viability and gene expression with combinations of multiple filamentous fungal species and cultivated meat relevant muscle cell types. We chose to work with bovine satellite cells (bSC) suitable for cultivated meat. Moreover, the inclusion of cell seeding conditions and differentiation compatibility extends our work’s impact. In short, this mycelium carrier technology can provide nutrition, flavor, and versatility for cultivated meat product development.

## Results

### Fungal species impact C2C12 attachment on mycelium carriers

**Fig. 1:**
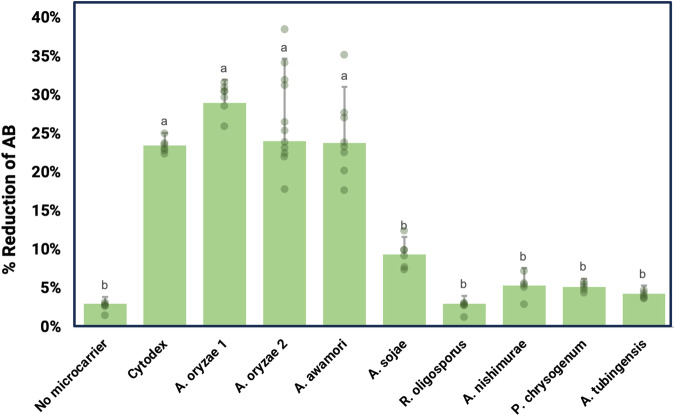
C2C12 metabolic activity expressed as percent reduction of alamarBlue (AB) on different fungal species at 48 h after seeding. No microcarrier as the negative control and nonedible carrier Cytodex as the positive control. Superscript letters (a–c) indicate statistically significant homogenous groups differing in the parameters among the strains (*p* < 0.05, *F*-test). Error bars represent standard deviations.

Different fungal species were made into mycelium carriers under the same fermentation conditions, then inactivated and seeded with C2C12 cells to observe the impact of fungal species on mycelium-cell interactions. Both food grade and non-food grade fungi were selected to broaden the range of species that may be compatible with animal cell attachment and growth (Table 1). Fungal morphology differed between species even when cultured under the same conditions due to their genetic differences (Table 1). Species such as *Rizosporus oligosporus* did not form spherical pellets but produced a matted structure, while other species spontaneously formed differently-sized pellets. Pellet diameter ranged from 1101 ± 580 μm for *A. oryzae* 1 and to 3050 ± 17 μm for *A. oryzae* 2. Carrier concentrations were selected so that all conditions had similar approximate available surface area per well, but all metabolic activity results were normalized to the actual calculated surface area. The metabolic activity of cells, expressed as percent reduction of alamarBlue (AB), was assessed at 48 hours after seeding, when C2C12 cells have attached to microcarriers (Fig. 1) and are in the exponential phase of growth. Any cells that did not attach have very low metabolic activity, which can be seen in the negative control condition (no microcarriers) where cells are forced to grow in suspension. Cytodex 3, a non-edible commercial microcarrier, was used for comparison. When used as carriers, different fungal species impacted C2C12 metabolic activity. C2C12 cells on *A. oryzae* 1, *A. oryzae* 2, and *A. awamori* showed strong reduction in AB: 29.00 ± 0.03%, 24.06 ± 0.11%, and 23.80 ± 0.07%, respectively. *R. oligosporus* (3.00 ± 0.01%), *A. nishimurae* (5.38 ± 0.02%), *P. chrysogenum* (5.19 ± 0.01%), and *A. tubingensis* (4.29 ± 0.01%) did not support C2C12 metabolism and were not significantly different from the negative control—implying that these species did not support cell metabolic activity most likely because cells did not attach to these carriers. *Aspergillus sojae* at 9.4 ± 0.02% reduction in AB suggested weak cell attachment, but the metabolic activity was less than half that of C2C12 cells on Cytodex. Therefore, *A. oryzae* 1 was chosen for subsequent experiments.

**Table 1:** Filamentous fungal species used to create carriers and their morphological characteristics

Strain	Strain Details	Morphology	Pellet Diameter (μm)	Origin
FST 76-2	*Aspergillus oryzae (A. oryzae 1)*		1101 ± 580^c^	UC Davis Phaff Culture Collection, Davis, CA, USA
UCD 8	*Aspergillus oryzae (A. oryzae 2)*		3050 ± 17^a^	UC Davis Viticulture and Enology Culture Collection, Davis, CA, USA
FST 40-400	*Aspergillus awamori*		2990 ± 650^a^	UC Davis Phaff Culture Collection, Davis, CA, USA
FST 76-1	*Aspergillus sojae*		1720 ± 427^bc^	UC Davis Phaff Culture Collection, Davis, CA, USA
UCD15	*Aspergillus nishimurae*		1200 ± 78^c^	UC Davis Viticulture and Enology Culture Collection, Davis, CA, USA
UCD12	*Aspergillus tubingensis*		2097 ± 162^b^	UC Davis Viticulture and Enology Culture Collection, Davis, CA, USA
FST 72-2	*Rhizopus oligosporus*		-	UC Davis Phaff Culture Collection, Davis, CA, USA
H3	*Penicillium chrysogenum*		2270 ± 71^b^	University of Cordoba, Department of Microbiology, Spain

Superscript letters (a–c) indicate statistically significant homogenous groups differing in the parameters among the strains (*p* < 0.05, *F* test). Scale bar is 1 cm.

### C2C12 proliferation is impacted by seeding concentration for mycelium carriers

**Fig. 2:**
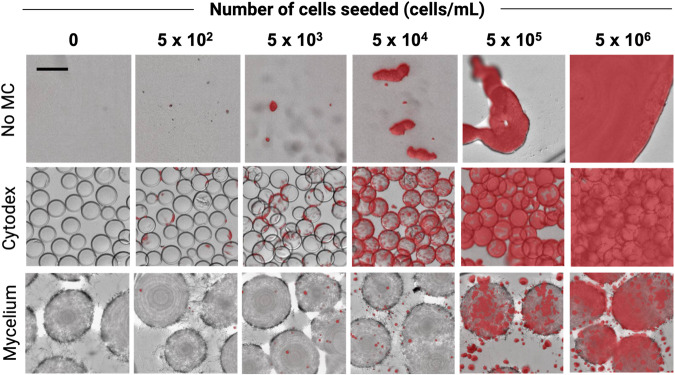
C2C12 on carriers at differing seeding densities. C2C12 transfected with TdTomato (red fluorescent) on Cytodex, Mycelium carrier (inactivated *A. oryzae* 1), and no microcarrier (MC) at 72 hours after seeding. Scale bar is 281 μm.

**Fig. 3:**
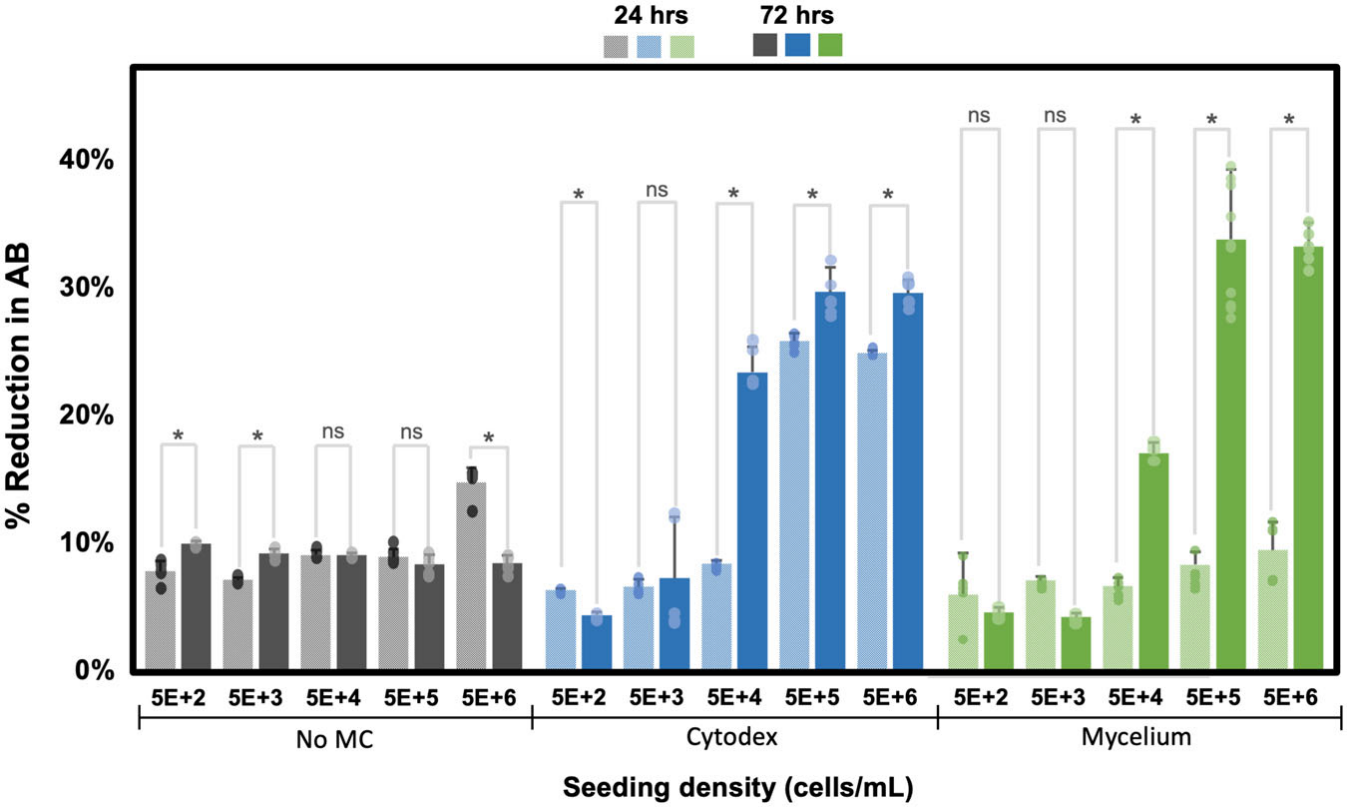
C2C12 metabolic activity represented as % reduction of alamarBlue (AB) on Cytodex, mycelium carriers and no microcarrier (MC) at 24 h from time of seeding (represented in lighter colored bars) and 72 h from time of seeding (represented in darker color bars). * indicates significant differences at *p* ≤ 0.05 with *n* ≥ 3 and ns indicates no significant differences. Error bars represent standard deviations.

To assess optimal seeding densities, carriers were seeded with C2C12 cells at concentrations of 5 × 10^2^, 5 × 10^3^, 5 × 10^4^, 5 × 10^5^, and 5 × 10^6^ cells/mL, and percent reduction of AB was measured at two time points (24 h and 72 h after seeding) to determine cell proliferation after 48 h in culture. Fluorescent microscope images of TdTomato-transfected C2C12 cells were taken at 72 h to visualize cells on carriers or formation of cell clumps (Fig. 2). When C2C12 cells are forced to grow in suspension, they aggregate with each other at concentrations of 5 × 10^4^ cells/mL and above. In the non-edible Cytodex microcarrier condition, C2C12 cells can be seen to evenly cover the entire surface of the beads. These high-density conditions showed saturation of C2C12 cells on the Cytodex beads where C2C12 were at 100% confluency in the 5 × 10^5^ cells/mL condition and formed large aggregates at the 5 × 10^6^ cells/mL condition. C2C12 cells cover the surface of the mycelium carriers at 5 × 10^6^ cells/mL with some small cell clumps attached to the strands of hyphae coming out of the center of the pellet. The group without microcarriers showed low AB percent reduction and little to no change in metabolic activity between the two timepoints except for the 5 × 10^6^ cell/mL condition, where some cells appeared viable at 24 h, but metabolic activity decreased from 15.7 ± 1.1% to 8.4 ± 0.6% AB reduction at 72 h (Fig. 3). Cytodex carriers showed the highest increase in metabolic activity at 5 × 10^4^ cells/mL seeding density, where percent AB reduction increased from 7.6 ± 0.2% to 21.3 ± 1.8%. Low-density seeding conditions (5 × 10^2^ and 5 × 10^3^ cells/mL) did not exhibit signs of proliferation with Cytodex and were lower in AB reduction or not significantly different from No MC conditions (Supplementary Fig. 1). High-density seeding conditions (5 × 10^5^ and 5 × 10^6^ cells/mL) showed sizeable AB percent reduction at the 24-h time point and modest proliferation over the course of 48 h. On the other hand, these high-density seeding conditions supported the largest increase in metabolic activity for C2C12 cells on mycelium carriers, increasing from 8.3 ± 1.0% to 33.7 ± 5.5% for 5 × 10^5^ cells/mL and 9.4 ± 2.2% to 33.2 ± 1.9% for 5 × 10^6^ cells/mL. At a seeding density of 10^4^ cells/well, C2C12 cells proliferated, albeit not as well, with 6.6 ± 0.7% AB reduction at 24 h and 17.0 ± 0.8% at 72 h. Low-density seeding conditions (5 × 10^2^ and 5 × 10^3^ cells/mL) did not exhibit signs of proliferation on mycelium carriers.

### C2C12 on mycelium carriers express differentiation markers

**Fig. 4:**
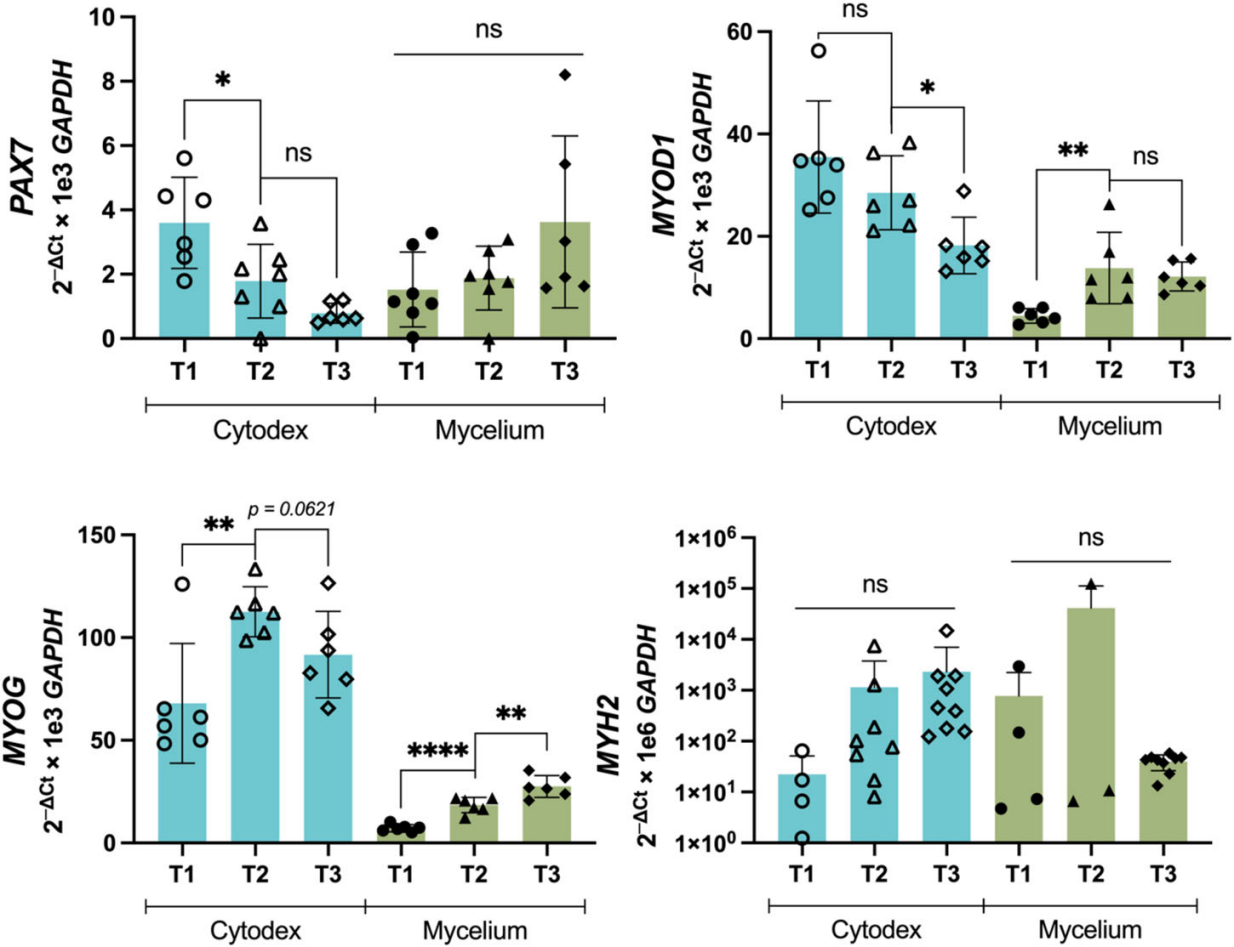
Relative expression of differentiation markers *PAX7*, *MYOD1*, *MYOG*, *MYH2* for C2C12 at four different sampling time points on cytodex and mycelium carriers. Samples were collected right after switching to differentiation media (time point 0, T0) and at 48 hours (time point 1, T1), 96 h (time point 2, T2), and 168 h (time point 3, T3). * indicates significant differences at *p* ≤ 0.05, ** at *p* ≤ 0.01, **** at *p* ≤ 0.0001, with *n* indicated by individual data points and ns indicating no significant differences. Plate wells that did not amplify are not shown. Error bars represent standard deviation.

Cell differentiation on microcarriers is an integral part of the cultivated meat production process, especially if the microcarriers are edible and cells remain on the carriers until the final product formulation. As a measure of the capacity of cells to differentiate on microcarriers, C2C12 cells on microcarriers were placed in differentiation medium for 7 days, and myoblast maturation markers, *PAX7*, *MYOD1*, *MYOG*, and *MYH2* were studied at three time points (Fig. 4). *PAX7* gene expression (associated with proliferation) decreased significantly from T1 to T2 for C2C12 on Cytodex carriers, while expression was detected but did not change significantly across timepoints on mycelium carriers. *MYOD1* expression showed a decreasing trend with time on Cytodex with significant decrease between T2 and T3. For mycelium however, *MYOD1* expression increased significantly between T1 and T2 and remained approximately the same at T3. *MYOG* expression fluctuated for Cytodex, increasing from T1 to T2 then decreasing slightly at T3. On the other hand, *MYOG* expression for cells grown on mycelium was less strong but showed steady and significant increases at each time point. Lastly, *MYH2* expressed strongly across all time points and with a substantial magnitude range, reflected using a logarithmic y-axis. Therefore, *MYH2* expression did not change significantly in the case of Cytodex or mycelium. Overall, differentiation gene expression was present, indicating C2C12 can differentiate on mycelium carriers.

### bSC attached and proliferated on mycelium carriers

**Fig. 5:**
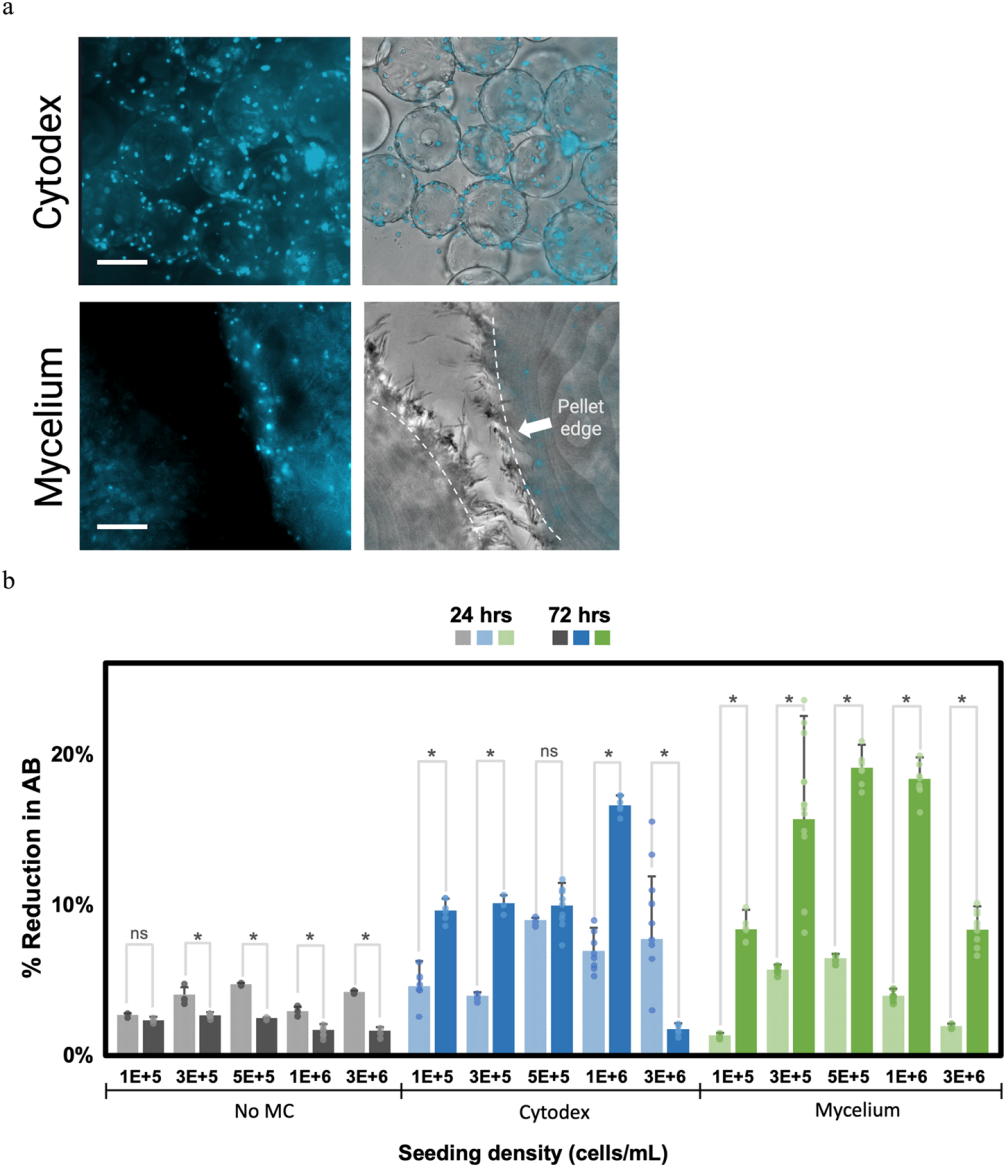
Bovine satellite cells on carriers. **a** bSC immunostained with Hoechst (blue fluorescent) on Cytodex microcarriers and mycelium carriers after 72-h incubation seeded at 5 × 10^5^ cells/mL. Dotted lines represent the edge of the pellet (mycelium carriers). **b** bSC metabolic activity represented as % reduction of alamarBlue (AB) on Cytodex, mycelium carriers and no microcarrier (MC) at 24 h from time of seeding (represented in lighter colored bars) and 72 h from time of seeding (represented in darker color bars). * indicates significant differences at *p* ≤ 0.05 with *n* ≥ 3 and ns indicates no significant differences. Error bars represent standard deviations. Scale bar is 130 μm.

To test the biocompatibility of mycelium carriers with relevant cell types, bSC attachment and proliferation was assessed. bSC are anchorage-dependent cells, as evidenced by the low metabolic activity when they are forced to grow in suspension and high metabolic activity when cells are anchored to carriers. bSC attachment on Cytodex and mycelium carriers can be confirmed with microscopy images (Fig. 5a). Proliferation of bSC was assessed by determining the bSC metabolic activity at 24 h and 72 h after seeding and seeding densities ranging from 1 × 10^5^ to 2.5 × 10^6^ cells/mL (Fig. 5b). bSC on Cytodex proliferated most when seeded at 1 × 10^6^ cells/mL, where percent reduction of AB increased from 7.0 ± 0.2% at the 24-h time point to 16.7 ± 0.6% at the 72-h time point. Cells on mycelium carriers proliferated at all seeding densities. At the 24-h time point, mycelium carriers had similar AB reduction as no microcarrier conditions except for 3 × 10^6^ cells/mL (Supplementary Fig. 2). However, AB reduction increased significantly at the 72-h time point, surpassing Cytodex conditions in some cases. Cells on mycelium carriers reached AB percent reduction higher than Cytodex for 5 × 10^5^ and 1 × 10^6^ cells/mL at T3; the greatest change was seen at the 1 × 10^6^ cells/mL condition where AB percent reduction increased from 4.0 ± 0.5% to 18.4 ± 1.4%.

### bSC on mycelium carriers express some differentiation markers

**Fig. 6:**
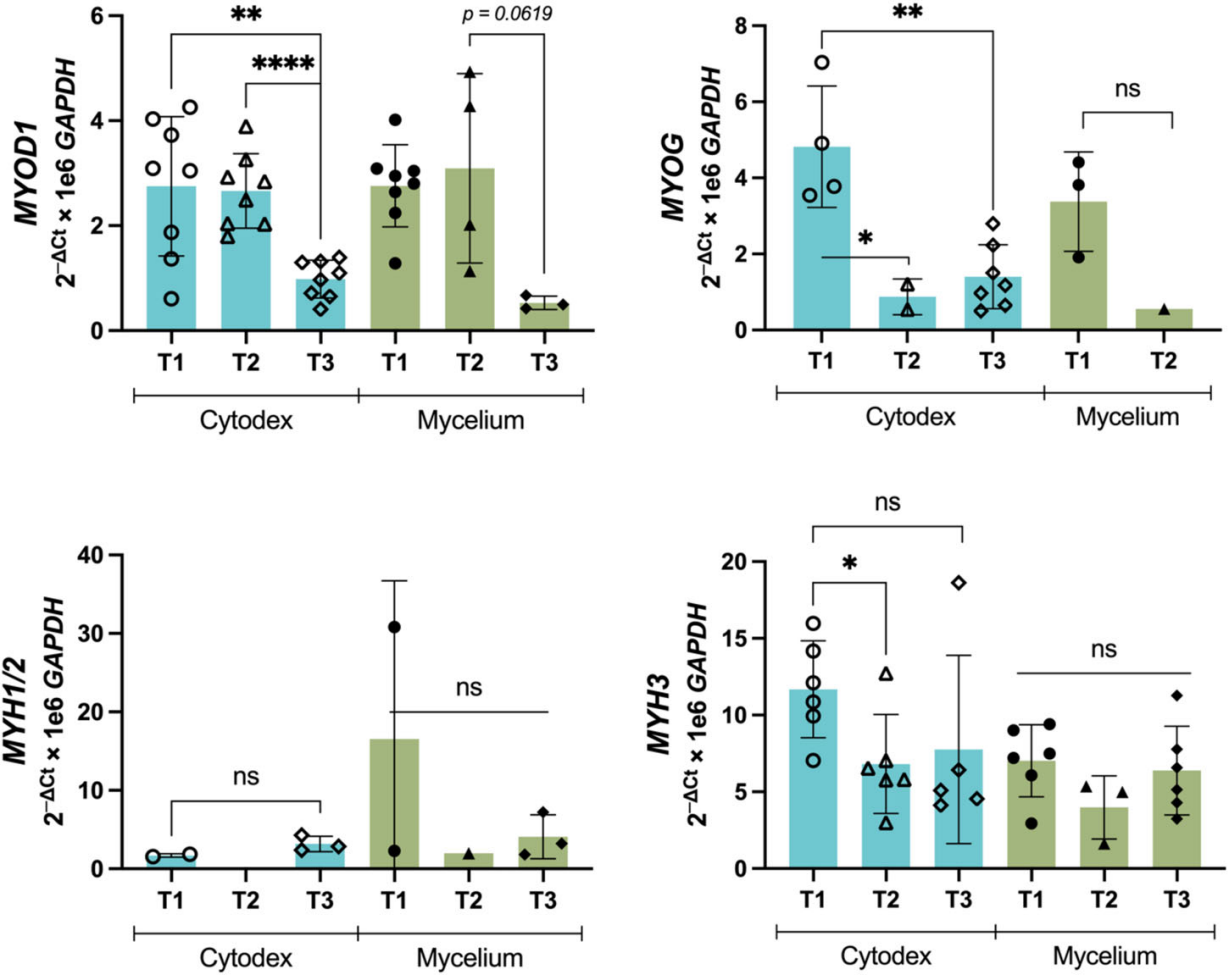
Relative expression of differentiation markers *MYOD1*, *MYOG*, *MYH1/2*, and *MYH3* for bSC at three time points on Cytodex and Mycelium carriers. Samples were collected 48 h (time point 1, T1), 96 h (time point 2, T2), and 168 h (time point 3, T3). * indicates significant differences at *p* ≤ 0.05, ** at *p* ≤ 0.01, **** at *p* ≤ 0.0001, with *n* indicated by individual data points and ns indicates no significant differences. Plate wells that did not amplify are not shown. Error bars represent standard deviation.

Similar to C2C12, bSC differentiation capacity on microcarriers was evaluated at three time points over 7 days following a change from proliferation to differentiation media. Gene expression associated with myogenic differentiation was studied using *MYOD1*, *MYOG*, *MYH1/2*, and *MYH3* (Fig. 6). Bovine satellite cell expression of *MYOD1* decreased over time on both Cytodex and mycelium carriers, though the expression of genes tested was not significantly reduced. *MYOG* expression also decreased over time for bSC on Cytodex from T1 to T3. Expression of *MYOG* was weak and not significantly different for bSC cells on mycelium carriers for T1 and T2 with no detectable expression at T3. Several samples expressed *MYH1/2* on both Cytodex and mycelium carriers, though like with *MYOG*, many samples failed to amplify. However, *MYH3* expression was clearly detected for cells on both Cytodex and mycelium carriers. The only significant difference is a reduction from T1 to T2 on Cytodex. Therefore, observed differentiation gene expression indicates that bSC can differentiate on mycelium carriers.

## Discussion

In this study, we demonstrated the potential of mycelium carrier technology by surveying several fungal species that can be compatibly used to culture cells relevant to cultivated meat. We further tested the differentiation capacity of cells on mycelium carriers, which is an important feature of edible carriers for cultivated meat production. Our results demonstrate wide utility of mycelium carrier technology, advancing our previously published POC and corroborating the carrier’s mammalian cell biocompatibility^21^.

Animal cells have differential metabolic activity and attachment in response to fungal species variation. The cell-carrier interface largely governs cell attachment. Surface hydrophobicity, charge, stiffness, elasticity, size, shape, topography and chemical composition are also known characteristics of cell-surface interface that impact the ability of a cell to anchor (reviewed in ref. ^6^). We saw that different fungal species yielded different sized pellets. This was expected because the genetic makeup of the species determines mycelium morphology^26,31^. We also observed that different strains produced more or less compact pellets (data not shown). This implies that surface topography, stiffness and elasticity is likely to differ between strains, thereby impacting attachment. In a previous study, we observed that heat-treated mycelium carriers showed better proliferation than chemical-treated mycelium carriers^21^. The heat-treated carriers weighed less and were slightly more hydrophobic than chemical-treated carriers. The impact of fungal species on attachment was also reported by Antinori et al., where two fungi of different genus were made into scaffolds to attach human dermal fibroblasts^32^. The authors speculated that residual extracts or fungal metabolites (which may be beneficial or toxic to cells) could be secreted, impacting cell attachment and viability. Our goal in this targeted strain screening was to select food-related fungal strains that were biocompatible with C2C12 cells, then move forward with the strain that showed the most promise for cell proliferation and differentiation. One limitation to our analysis is that it fails to quantitatively measure available area for cell attachment on fungal pellets. Numerous attempts were made to quantify fungal pellet specific surface area, without any improvement on the smooth spherical pellet assumption. Thus, we did not quantitatively characterize fungal strain topology in detail. Our focus was on the efficacy of this edible and sustainable biomaterial to support mammalian skeletal muscle culture. In this study, our results show that fungal microcarriers perform promisingly and could be used in alternative protein products. To improve this technology, it is necessary to adapt it to specific cell culture practices and characterize fungal pellet strains in detail, including specific surface area analysis. A characterization dataset for fungal strains can inform high-throughput screening that aligns cell types with compatible fungal strains. Moreover, optimizing the technology involves fine-tuning process parameters like dissolved oxygen control, pH control, and nutrient feeds to enhance productivity of animal cell-mycelium carrier systems and maximize cell density in the media^33,34,35^.

For mycelium carriers, seeding density optimization clearly showed a benefit to a high seeding density (5 × 10^5^ and 5 × 10^6^ cells/mL), resulting in better C2C12 cell proliferation. Seeding optimization is commonly performed in cell culture, since cell-cell contact inhibition is a factor that impacts cell signaling for proliferation and other biological cascades^36^. The same high cell densities for Cytodex and suspension (no microcarrier) culture systems resulted in large aggregates with low metabolic activity. In many cases, large (>500 µm diameter) cell aggregates are difficult to control because heterogeneous cell clumps and mass transfer limitations yield necrotic cores^37,38^. On the other hand, small aggregates—which are not limited by diffusion—can maintain cell viability. Norris et al.^39^ showed that small cell aggregates, named microtissues, support myogenic cell growth and differentiation, and can be a promising solution to address scalability of cultivated meat production at bioreactor scale. For mycelium carriers, fluorescence images showed numerous small aggregates surrounding the surface of the mycelium carriers. These aggregates were much smaller than those observed in suspension (no microcarrier) and Cytodex conditions, and they did not clump into large aggregates even at high cell seeding densities. These findings suggest that mycelium carriers have potential to control aggregate size and could be used for scalable cultivated meat production using a single bioreactor. It also suggests a strong interaction between the cells and the Cytodex surface, and a weaker interaction between the cells and the mycelium surface. Cadherin-mediated cell clumping on mycelium may enable the fungal filaments to entrap cell aggregates near the pellet surface.

Further, we demonstrated that C2C12 on fungal carriers expressed key markers of myogenesis in response to media-induced differentiation. *MYOD1*, *MYOG*, and *MYH* are myogenic regulatory factors that regulate the development of functional embryonic skeletal fiber muscle differentiation^40,41^. *MYH1* and *MYH2* are genes that encode MyHC-2X and MyHC-2A, respectively. Both proteins are found in fast-twitch adult skeletal muscle. *MYH3* encodes MyHC-emb, an embryonic skeletal muscle protein^42^. While the expression of these transcription factors functions as a switch in the differentiation of myoblasts, *PAX7* is a proliferation regulator of muscle stem cells and is activated during muscle regeneration^43^. We observed increases in expression of *MYOD1* and *MYOG* over time and expression of *MYH2* for C2C12 on mycelium carriers. The expression of these key markers, especially the strong expression of *MYH2*, indicate that C2C12 are differentiating on microcarriers such as Cytodex^44^. Interestingly, *PAX7* expression was observed not to significantly decrease on mycelium carriers over time indicating that some C2C12 cells may still be maintaining their proliferative state while others are differentiating. Compared to the smooth, monolayered surface of Cytodex, the mycelium is structurally intricate and compositionally complex, consisting of a porous matrix of various protein, glucans, and chitin. *MYH2* expression is detectable, though the wide range of expression renders no comparison significantly different. Nevertheless, we consistently detected late myogenic gene expression in both the Cytodex and mycelium carriers.

Since the focal application of this technology is cultivated meat production, it was imperative to demonstrate cell growth potential using a cell type suitable for cultivated meat. We chose bSC, which can proliferate and maintain stemness. Satellite cells are mononuclear cells located between the basal membrane and the sarcolemma of adjacent muscle fibers in mammalian skeletal muscles^45^. Unlike embryonic stem cells, induced pluripotent stem cells, and mesenchymal stem cells that have multilineage differentiation capacity, satellite cells are lineage-restricted and differentiate into myocytes upon activation from quiescence^46^. This unique property makes them ideal for facilitating the bioprocess of muscle cell production. Our results demonstrate bSC attachment to mycelium carriers and increased proliferation compared to Cytodex at the same seeding density. Trends in differentiation gene expression were difficult to assess. However, *MYOD1* and *MYH3* expression was clear across all sampling points. This, coupled with the fact that *PAX7* expression was undetectable, suggests that spontaneous differentiation may be occurring early on and stemness of bSC is lost perhaps even before differentiation was induced by media change. Nonetheless, bSC appear to have similar differentiation potential on mycelium as they do on Cytodex carriers. These are promising initial observations that a one-step proliferation/differentiation bioprocess on edible microcarriers may be possible. In cultivated meat, such a simplified process where the microcarrier formation, cell proliferation, and cell differentiation could occur in a single bioreactor is highly advantageous. Such a process would reduce manufacturing time, contamination risk, and the cost for other specialized equipment. The bSC cell type is also favorable, as they have been observed to proliferate in spinner flasks on Cytodex and edible carriers composed of gelatin and eggshell membrane with collagen^39,47^. Verbruggen et al.^48^ demonstrated bovine myoblast proliferation and differentiation on Cytodex in spinner flasks. Thus, we further hypothesize that bSC on mycelium carriers have potential to grow and differentiate in culture with agitation at larger scales.

In summary, we were able to demonstrate the applicability of mycelium carriers for cultivated meat production. While the experiments of this study were limited to microwell plates in static conditions, the data presented here are necessary foundational knowledge to fuel future investigations of this edible biomaterial for cellular agriculture applications. For example, future and ongoing work to advance the technology can tailor mycelium fermentation and cell culture conditions to the respective cell type and enhance productivity. Scaling up cell culture to larger volumes and validating bead-to-bead transfer under agitation conditions represent immediate, evident progressions. Investigating the impact of mycelium and different strains of fungi on the flavor, texture, and nutritional attributes of the final cultivated meat or seafood remains a valuable avenue for product refinement and development. Furthermore, leveraging biological organisms like filamentous fungi as biomaterials holds promise not only due to their edible qualities but also their genetic modifiability. The use of genetic modification in cultivated meat is still uncertain, however, engineering filamentous fungi is technically feasible. Using this approach, we can create mycelium carriers superior at facilitating animal cell proliferation and differentiation or amplify sensory attributes to be more appetizing. Even if not directly applicable to cellular agriculture, this avenue of research contributes to comprehending the fundamental science underpinning cell-mycelium interactions and can find applications in sectors like medical and pharmaceutical cell culture, where genetic modification is less restrictive.

## Methods

### Fungal strains and media components

Filamentous fungal strains were obtained from the UC Davis Viticulture and Enology Department Culture Collection (Davis, CA, USA), UC Davis Phaff Yeast Culture Collection (Davis, CA, USA), and the University of Cordoba Culture Collection (Cordoba, Spain). The strains are *Aspergillus oryzae* FST 76-2, *Aspergillus oryzae* UCD 8, *Aspergillus awamori* FST 40-400, *Aspergillus sojae* FST 76-1, *Aspergillus nishimurae* UCD 15, *Aspergillus tubingensis* UCD 12, *Rhizopus oligosporus* FST 72-2, and *Penicillium chrysogenum* H3 (Table 1).

Filamentous fungi were pre-grown on a common sporulation agar media (1.7% (*w*/*v*) corn meal agar (BD, Franklin Lakes, NJ, USA), 0.1% (*w*/*v*) yeast extract (BD, Franklin Lakes, NJ, USA), 0.2% (*w*/*v*) glucose (Spectrum Chemicals, New Brunswick, NJ, USA) and 2% (*w*/*v*) bactoagar (BD, Franklin Lakes, NJ, USA)) and filamentous fungal pellet medium (6% (*w*/*v*) glucose, 0.3% (*w*/*v*) yeast extract, 0.3% (*w*/*v*) NaNO_3_ (Sigma-Aldrich, St. Louis, MO, USA), 0.1% (*w*/*v*) K_2_HPO_4_ (Thermo Fisher Scientific, Waltham, MA, USA), 0.05% (*w*/*v*) MgSO_4_, (RPI, Mount Prospect, IL, USA) 0.05% (*w/v*) KCl (Thermo Fisher Scientific, Waltham, MA, USA), and 0.001% (*w*/*v*) FeSO_4_ (Thermo Fisher Scientific, Waltham, MA, USA). Inactivation check was performed on YPD plates (yeast extract 1% (*w*/*v*), peptone 2% (*w*/*v*), glucose 2% (*w*/*v*), bactoagar 2% (*w*/*v*)).

### Animal cells and culture media

C2C12 cells (mouse myoblasts; ATCC: CRL-1772) and bovine satellite cells (bSC) were studied for proliferation and differentiation potential on microcarriers. To aid with microscopy, C2C12 cells were transduced with tdTomato (C2C12 TD) using a *Lentivirus* construct. Lentiviral particles encapsulating tdTomato-encoding plasmid were acquired from the UC Davis Health Vector Core. Approximately 10^5^ C2C12 cells were incubated with lentivirus at a multiplicity of infection (MOI) of ∼7.5 for 24 h. Cells were expanded after clonal selection of tdTomato-positive cells, using a cloning cylinder and validated for reporter gene expression by fluorescence miscroscopy^49,50^. C2C12 cells were then cultivated in complete proliferation media: 89% (*v*/*v*) DMEM (Thermo Scientific, Rochester, NY, USA), 10% (*v*/*v*), fetal bovine serum (Thermo Scientific, Rochester, NY, USA), 1% (*v*/*v*) Penicillin-Streptomycin (Sigma-Aldrich, St. Louis, MO, USA) and differentiation media: 97% (*v*/*v*) DMEM (Thermo Scientific, Rochester, NY, USA) 2% (*v*/*v*), horse serum (Thermo Scientific, Rochester, NY, USA), 1% (*v*/*v*) Penicillin-Streptomycin (Sigma-Aldrich, St. Louis, MO, USA). bSC were isolated from fresh leg skeletal muscle of XX/XXY Holstein Cow at Animal Science Department the University of California, Davis. Briefly, the biopsy was cut into 0.5 cm or smaller bundles and digested with 2% penicillin/streptomycin/amphotericin (Lonza 12-745E) and 200 units/mL collagenase (Worthington, Lakewood, NJ, USA) for 2 h. Debris was removed by repeated centrifugation, washing, and filtering through a 100 µm filter and 40 µm filter. Cells were incubated with an FcR blocking reagent, labeled with micromagnetic brands in the satellite cell isolation kit (Miltenyi), and passed through a MACS column and separator (Miltenyi) with bSC sorted out via negative selection. bSC was cultivated in complete proliferation media: 79% (*v*/*v*) F10 (Thermo Scientific, Rochester, NY, USA), 20% (*v*/*v*) fetal bovine serum (Thermo Scientific, Rochester, NY, USA), 1% (*v*/*v*) Penicillin-Streptomycin (Sigma-Aldrich, St. Louis, MO, USA) supplemented with 5 ng/mL FGF2 (Thermo Scientific, Rochester, NY, USA) and differentiation media: 93% (*v*/*v*), DMEM (Thermo Scientific, Rochester, NY, USA), 2% (*v*/*v*) fetal bovine serum (Thermo Scientific, Rochester, NY, USA), 1% (*v*/*v*) Penicillin-Streptomycin (Sigma-Aldrich, St. Louis, MO, USA).

### Mycelium carrier formation and inactivation

All fungi used in this study were grown under the same conditions as the initial POC^21,22^. Filamentous fungal strains were pre-grown on sporulation agar plates for 7 days at 28 °C. Conidia and spore formation was confirmed via confocal and light microscope. The spores were then suspended in sterile distilled water and vortexed, then sonicated for 5 min. The suspension was inoculated into 50 mL of filamentous fungal pellet medium, adjusted to a pH of 5.5, in 250 mL Erlenmeyer flasks covered with hydrophobic cotton, with the final spore concentration reaching 1 × 10^6^ spores/mL. The flasks were placed under high agitation at 250 rpm and a temperature of 30 °C in an Innova 4000 Incubator Shaker (New Brunswick Scientific Co., Edison, New Jersey). This produced spherical, porous biomasses, called pellets (except *R. oligosporus*). These pellets spontaneously form with certain species of fungi when cultivated in this condition. After 3 days, the fungal pellets were harvested, washed with sterile DI water, then immediately autoclaved in sterile DI water at a temperature of 121 °C for 20 min. Inactivation was confirmed by taking 20 random pellets and assessing growth on YPD agar plates at 28 °C for at least 1 week. No growth was observed, which confirmed that the pellets were inactive. Brightfield images of the pellets were characterized using the particle analysis feature in FIJI (Bethesda, Rockville, MD, USA)^51^.

### Biocompatibility, initial seeding concentration, and proliferation potential

Several species of mycelium carriers were screened for biocompatibility with C2C12 cells. Carriers were pretreated by washing twice with Dulbecco’s phosphate-buffered saline (DPBS) (Gibco, Thermo Fisher Scientific, Waltham, MA, USA) and submerging in complete proliferation media for 3 h prior to seeding. Cytodex 3 (Cytiva, Marlborough, MA, USA), the commercial standard non-edible carrier, was used as a positive control. Cytodex 3 are non-porous, type I porcine collagen-coated beads, composed of cross-linked dextran and a diameter of 141–211 µm and 2700 cm^2^/g dry weight specific surface area. Cytodex 3 was prepared at 3 g/L per replicate then hydrated and pre-treated according to the manufacturer’s instructions before seeding. Mycelium carrier conditions were standardized by assessing fungal strain differences in diameter and approximating to match the available surface area to that of the Cytodex conditions. One strain in particular, *R. oligosporus*, did not form spherical pellets but a flat, matt-like structure. For this condition, a circular cutout with ~7 mm diameter and 1 mm thickness was placed at the bottom of the 96 well with cells seeded above it for biocompatibility testing. After pretreatment of carriers, the C2C12 cells were seeded in 96-well plates coated with anti-adherent solution (StemCell Technologies, Vancouver, BC) to prevent them from anchoring to the bottom of the wells. Negative control wells had no microcarriers. Wells that contained only media or only microcarriers were prepared as blanks. Each replicate had its own dedicated well on the 96-well plates. The cells were incubated at 37 °C with 5% CO_2_ in a HERAcell VIOS 160i incubator (Thermo Fisher Scientific, Waltham, MA, USA).

alamarBlue® (AB) Cell Viability Assay Reagent (Invitrogen, Waltham, MA, USA) was used to determine the number of viable cells. This dye incorporates an oxidation-reduction indicator that fluoresces and changes color in response to chemical reduction within active mitochondria. A 20 μL AB solution was added to each replicate 48 h after seeding. After four hours of incubation, 100 μL of media with AB solution was transferred to a clear bottom 96-well plate, and absorbance was measured at 570 nm and 600 nm using the SpectraMax iD5 Multi-Mode Microplate Reader (Molecular Devices, San Jose, CA, USA). The percentage reduction of AB was calculated per the manufacturer’s instructions. Previous work from our lab has found that the higher the percentage reduction of media, the more metabolically active cells present^35^. Assuming a uniform (average) metabolic activity for each cell, this percent reduction is linearly correlated with cell concentration. This simple assessment gives us a way of comparing between groups with this proxy for biocompatibility and proliferation potential.

The fungal species, *A. oryzae* 1, which showed the highest percent reduction in AB, was chosen for a seeding concentration study. Cytodex and mycelium carriers were prepared as described above and each replicate used the same cell concentration. Seeding conditions for C2C12 cells were 5 × 10^2^, 5 × 10^3^, 5 × 10^4^, 5 × 10^5^, 5 × 10^6^ cells/mL and for bSC were 1 × 10^5^, 2.5 × 10^5^, 5 × 10^5^, 1 × 10^6^, 2.5 × 10^6^ cells/mL. At 24 and 72 h after seeding, the cell number was analyzed by AB following the methods described above.

### Differentiation potential

Differentiation potential of C2C12 cells on microcarriers was tested by taking carriers with cells at >90% confluence, washing twice with DPBS, and adding 200 μL of differentiation media. The cells were incubated at 37 °C with 5% CO_2_. The entire sample volume was collected at 48 h (T1), 96 hours (T2), and 168 h (T3) after switching to differentiation media. Each time-point replicate was a dedicated well on the 96-well plate coated with anti-adherent solution (StemCell, Vancouver, WA, Canada). Each time point was performed at least in triplicate.

The collected samples were immediately placed in TRIzol reagent (Invitrogen, Carlsbad, CA) and frozen at −80 °C. Defrosted samples were mixed with chloroform and RNA was separated from cell proteins and DNA in a liquid–liquid phase separation per manufacturer instructions. The RNA was precipitated and purified with IPA/EtOH/H_2_O and then re-dissolved in RNase-free water (Qiagen, Hilden, Germany). The concentration of RNA was measured with a NanoDrop (Thermo Fisher Scientific, Waltham, Massachusetts, USA) and diluted to a uniform concentration between 20 and 50 ng/µL. The extracted and purified RNA was reverse transcribed using Quantiscript Reverse Transcriptase, Quantiscript RT Buffer, and RT Primer Mix (Qiagen, Hilden, Germany) using the manufacturer’s instructions.

The TaqMan PCR reaction was performed in a final volume of 10 μL using 7 μL of Quantitect Probe PCR mix (Qiagen, Hilden, Germany), 30 ng cDNA, and 0.5 μL of each TaqMan probe with FAM reporter dye. The amplifications were performed on an Applied Biosystems™ QuantStudio™ 6 Pro Real-Time PCR System, 96-well, 0.1 mL block (Thermo Fisher Scientific, Waltham, Massachusetts, USA). The thermal cycling protocol was 95 °C for 3 min, followed by 45 cycles of 95 °C for 3 s and 60 °C for 30 s. Mouse-specific TaqMan Assays for *GAPDH* (Mm99999915_g1), *MYOD1* (Mm00440387_m1), *MYOG* (Mm00446194_m1), *MYH2* (Mm01332564_m1), and *PAX7* (Mm01354484_m1) were used. Bovine-specific TaqMan Assays for *GAPDH* (Bt03210913_g1), *MYOD1* (Bt03244740_m1), *MYOG* (Bt03258929_m1), *MYH1/2* (Bt03223147_gH), and *MYH3* (B03258391_m1). Rabbit *PAX7* (Oc06751922_s1) was used for bSC differentiation analysis because of a lack of bovine-specific assay and likely cross-species reactivity, but no expression was observed. All TaqMan Assays were purchased from Thermo Fisher Scientific. Results were normalized to the corresponding sample’s endogenous control, *GAPDH*. Reactions were thrice replicated and plate wells that did not amplify were not plotted. The data are presented in Fig. 4 and Fig. 6 as the single delta fold-change in gene expression compared to *GAPDH* × 1e3 or 1e6, depending on the gene of interest.

### Microscope imaging analysis

Fluorescent microscopy was used to visualize cells on Cytodex and mycelium carriers as well as in suspension. For C2C12 cells, the myoblasts were transfected with tdTomato and imaged in the TRITC channel with a 10× objective. Transfection methods are described in ref. ^50^. bSC were immunostained with Hoechst 33342 (1:2000; Invitrogen A32766) and imaged in the DAPI channel with a 20× objective. All samples were imaged using an ImagePICO (Molecular Devices, San Jose, CA, USA) with z-stack.

### Statistical analysis

Statistical analysis was performed using the software R (v4.2.1; R Core Team, 2022) and the *agricolae* package (v1.3-5; de Mendiburu, 2021) for the following methods: ANOVA (Analysis of Variance) and Fisher’s LSD test for the establishment of homogeneous groups (HG) and two-tailed unpaired *t*-test. Any statistically significant difference was implied by *p* ≤ 0.05 unless otherwise stated in the figures. At least three biological replicates were performed for every condition and time point, with each biological replicate corresponding to a dedicated well on a 96-well plate.

### Reporting summary

Further information on research design is available in the Nature Research Reporting Summary linked to this article.

### Supplementary information

Supplemental Figures

Reporting Summary

## Data Availability

Data sets generated during the current study are available from the corresponding author upon reasonable request.
