# Supplementary material for: Edible mycelium as proliferation and differentiation support for anchorage-dependent animal cells in cultivated meat production
Source: NPJ Sci Food. 2024 Apr 30;8:23. doi: 10.1038/s41538-024-00263-0 (PMC11063153; doi:10.1038/s41538-024-00263-0)
Supplement: Supplementary file 1 — Supplemental Figures [file 41538_2024_263_MOESM1_ESM.pdf]

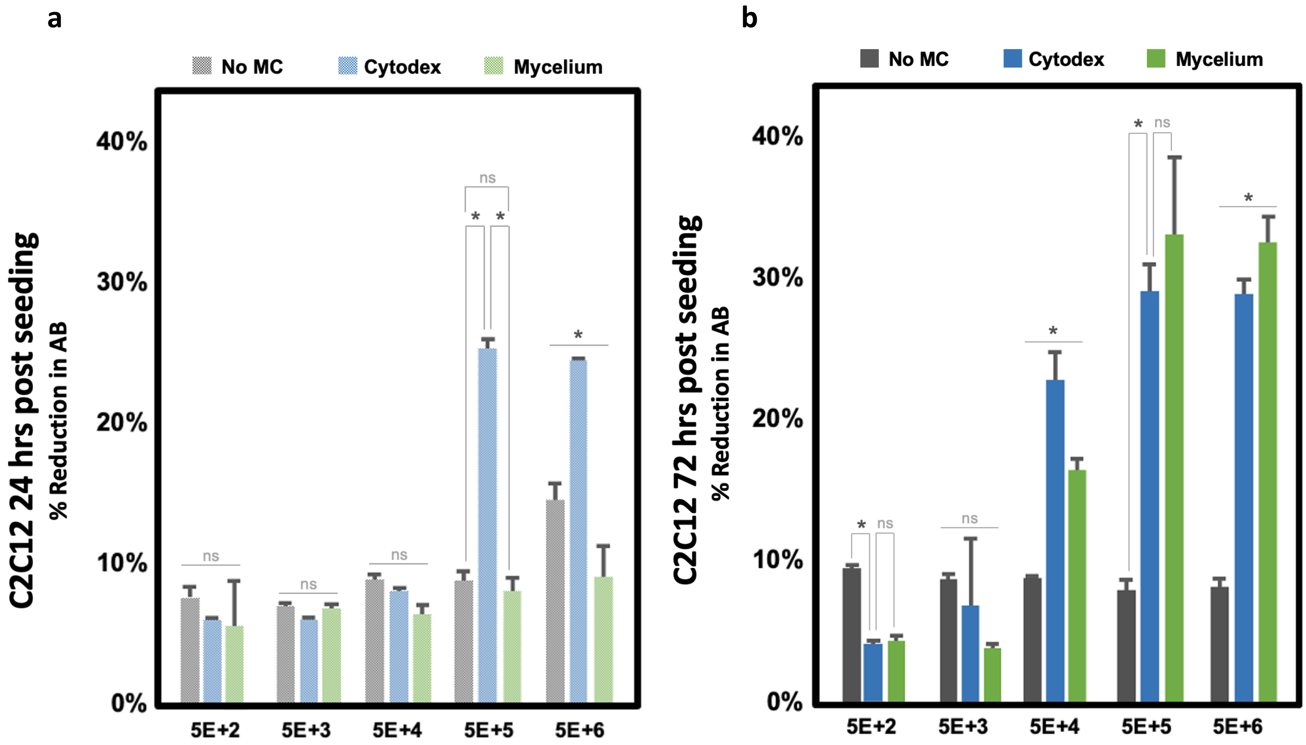

Supplementary Figure 1. C2C12 metabolic activity represented as % reduction of AlamarBlue (AB) on Cytodex, mycelium carriers and no microcarrier (MC) at (a) 24 hours from time of seeding, and (b) 72 hours from time of seeding. Error bars represent SD.
* indicates significant differences at $p \leq 0.05$ with $n \geq 3$ and ns indicates no significant difference

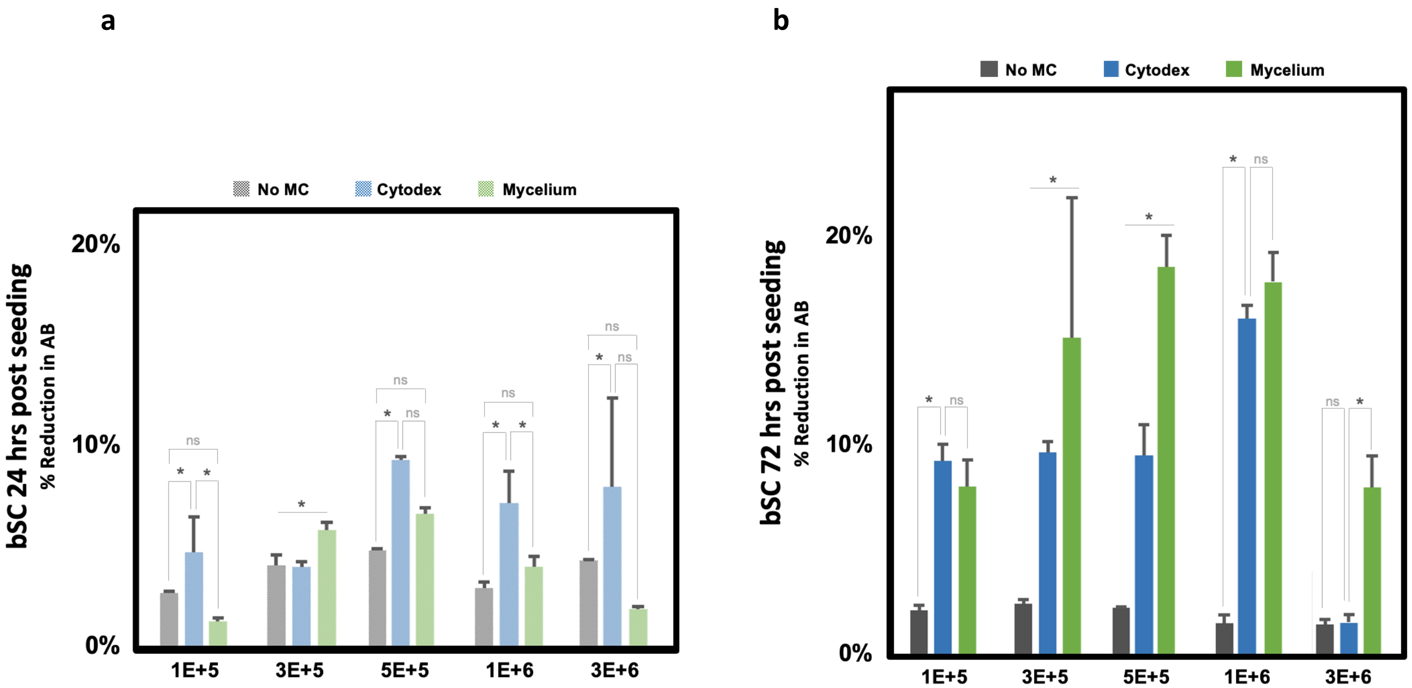

Supplementary Figure 2. bSC metabolic activity represented as % reduction of AlamarBlue (AB) on Cytodex, mycelium carriers and no microcarrier (MC) at (a) 24 hours from time of seeding, and (b) 72 hours from time of seeding. Error bars represent SD. * indicates significant differences at $p \leq 0.05$ with $n \geq 3$ and ns indicates no significant differences
